# Non-transport roles of nuclear import receptors: In need of the right balance

**DOI:** 10.3389/fcell.2022.1041938

**Published:** 2022-11-10

**Authors:** Michela Damizia, Ludovica Altieri, Patrizia Lavia

**Affiliations:** ^1^ Department of Cellular, Computational and Integrated Biology (CIBIO), University of Trento, Trento, Italy; ^2^ Institute of Molecular Biology and Pathology (IBPM), CNR National Research Council of Italy, Sapienza University of Rome, Rome, Italy; ^3^ Department of Biology and Biotechnology “Charles Darwin”, Sapienza University of Rome, Rome, Italy

**Keywords:** nuclear transport, importin beta family, mitotic spindle, nucleoporins, ubiquitin/proteasome system, protein aggregates, neurodegenerative diseases, ciliogenesis

## Abstract

Nuclear import receptors ensure the recognition and transport of proteins across the nuclear envelope into the nucleus. In addition, as diverse processes as mitosis, post-translational modifications at mitotic exit, ciliogenesis, and phase separation, all share a common need for regulation by nuclear import receptors - particularly importin beta-1 and importin beta-2/transportin - independent on nuclear import. In particular, 1) nuclear import receptors regulate the mitotic spindle after nuclear envelope breakdown, 2) they shield cargoes from unscheduled ubiquitination, regulating their timely proteolysis; 3) they regulate ciliary factors, crucial to cell communications and tissue architecture during development; and 4) they prevent phase separation of toxic proteins aggregates in neurons. The balance of nuclear import receptors to cargoes is critical in all these processes, albeit in opposite directions: overexpression of import receptors, as often found in cancer, inhibits cargoes and impairs downstream processes, motivating the therapeutic design of specific inhibitors. On the contrary, elevated expression is beneficial in neuronal contexts, where nuclear import receptors are regarded as potential therapeutic tools in counteracting the formation of aggregates that may cause neurodegeneration. This paradox demonstrates the amplitude of nuclear import receptors-dependent functions in different contexts and adds complexity in considering their therapeutic implications.

## Mechanistic paradigms for nuclear import receptors

Since the discovery that protein localisation depends on specific signal sequences (Blobel, 2000), nuclear transport receptors for such signals have been seen as global regulators of cellular organisation due to their ability to transport cargoes in and out of the nucleus, hence marking the identity of subcellular compartments.

Nuclear import receptors (NIRs) also play roles beyond transport. Here we will summarise our understanding of NIRs from nuclear import studies (Christie et al., 2016; Stewart, 2022; Wing et al., 2022), then examine how their mechanism(s) of action readapt to regulate processes independent on nuclear import, yet sensitive to regulation by NIRs: mitosis, mitotic exit, ciliogenesis, and phase separation.

Briefly, proteins to be imported within nuclei, carrying nuclear localisation signals (NLSs), are recognised by a receptor of the importin alpha family, which have subtle NLS sequence preference and tissue distribution (Kimura and Imamoto, 2014). NIRs of the importin beta family bind either the importin alpha/cargo dimer, or certain NLS proteins directly, and provide directionality to the complex. During transport, NIRs keep their interacting cargoes in a non-functional state, that will be reversed in the nucleus, where the GTPase RAN is activated by the chromatin-bound GTP exchange factor RCC1 (regulator of chromosomes condensation 1). Therein, RANGTP binding to the NIR dissociate the import complex and release functional NLS cargoes.

Nuclear import also relies on the interaction of NIRs with nuclear pore complexes (NPCs) that fenestrate the nuclear envelope (NE), providing gates to the nuclear periphery. About 30 nucleoporins (NUP) organise the NPC in a basket-shaped structure. Import complex “navigate” through the NPC central channel aided by the NUPs. Importin beta-1 interacts first with NUP358/RANBP2, the largest NUP, placed at the cytoplasmic base of the NPC. That interaction, directed by phenylalanine-glycine (FG)-rich regions of RANBP2 projecting from the NPC, helps orienting import complexes. Once attached to RANBP2, importin beta-1 interacts sequentially with FG-rich NUPs positioned along the NPC and forming a permeability barrier in nuclear pores (Bednenko et al., 2003). FG-rich domains are intrinsically disordered and can undergo phase separation, forming a dense meshwork that remains permeable to importin beta-type NIRs, but not to other macromolecules. The interaction of NIRs with FG domains enables the import complex passage through the NPC (Schmidt and Görlich, 2016). NUPs are therefore integral components of the nuclear transport machinery. The solubilisation of FG domains by NIRs indicates a widespread ability to disperse proteins in phase separations in different contexts (Yoshizawa and Guo, 2021). RANBP2 also has an additional function as a SUMO E3 ligase and SUMO-stabilising factor (Pichler et al., 2002). It interacts with the RAN GTPase-activating protein RANGAP1 and stabilises it in the SUMO-conjugated form, keeping SUMOylated RANGAP1 and the SUMO-conjugating enzyme UBC9 at the NPC base: this complex regulates the SUMOylation state of some transport cargoes (Ptak and Wozniak, 2017), and concomitantly determines a steep difference between the RANGTP-rich nucleus and the cytoplasm, which modulates entry and exit of transport complexes.

In proteomics studies, importin beta members have diversified partners and cargo preferences in different cell types, cell cycle phases and subcellular compartments (Roscioli et al., 2012; Kimura et al., 2013a, 2013b, 2017; Hugel et al., 2014; Mackmull et al., 2017; Baade et al., 2018; Di Francesco et al., 2018; Song et al., 2022). This indicates their potential to act in diverse cellular processes *via* their capacity to localise NLS-tagged cargoes and modulate protein interactions. The next sections summarise these processes and highlight how altered expression of NIRs can disrupt them, with pathogenetic consequences.

## Localisation and function: Re-adapting nuclear import receptors mechanisms from nuclear import to mitotic control

In mitosis, when nucleo-cytoplasmic transport ceases, NIRs localise a group of NLS factors, released from the nucleus at NE breakdown and regulating microtubule nucleation, interactions with kinetochores, and dynamic functions, collectively called spindle assembly factors (SAFs) (reviewed by Forbes et al., 2015; Cavazza and Vernos, 2016).

In interphase, the NE provides a physical barrier between subcellular compartments in which NIRs and RAN are differentially abundant. In mitosis, in the absence of any such barrier, spatial clues are critical in dictating the relative position of NIRs vs*.* RAN. Importin beta-1 interacts with the spindle microtubules and poles. RANGTP, generated by histone-bound RCC1, is abundant around chromosomes, including at kinetochores, where factors required for microtubule nucleation and kinetochore attachments are recruited. Centrosomes, the canonical microtubule-nucleation centers, also recruit a RAN fraction *via* the anchoring protein AKAP450. Thus, a topological map is established.

Regions of differential importin:RANGTP concentrations (high RANGTP around chromosomes, high NIRs at the mitotic apparatus) reorganise dynamically over varying distances during mitotic progression. During this time, the functional antagonism between NIRs and RANGTP continues to operate, and their relative abundance determines the functional state of SAFs at any given time and site in mitotic cells. Importin beta-1 keeps SAFs inactive while localising them, until chromosome- or centrosome-associated RANGTP binds to it and locally releases active SAFs (Roscioli et al., 2010). Importin beta-2 (transportin-1, TNPO1) also regulates SAFs *via* direct inhibition, with a binding mechanism that is partially RANGTP-reversible (Bernis et al., 2014). Thus, NIRs act as master regulators of the mitotic apparatus by governing the spatial programme of SAF activation.

After chromosome segregation has occurred, a coordinated dephosphorylation programme ensures chromatin decondensation and nuclear reassembly. The main actors at that stage are protein phosphatase 1 (PP1) and 2 (PP2A), representing the catalytic moiety of complexes that include regulatory or scaffolding subunits. Importin beta-1 plays roles in reconstituting the NE and resetting the interphase state and it interplays with both major phosphatases to achieve this programme (Table 1).

**TABLE 1:** Target factors of NIRs in non-import processes.

NIR	NIR cargo/ targeted process	NIR-interacting localisation signal(s) in cargo	NIR functions	Reference
Mitotic completion^a^				
Importin beta-1	HURP	NLS	Importin beta-1 binding prevents the release of HURP at its site of action (i.e. microtubule growing ends) and its premature interaction with APC/C.	Silljé et al. (2006), Song and Rape (2010), Song et al. (2014)
Importin beta-1	NuSAP1	NLS	Importin beta-1 binding prevents NUSAP1 function in formation of microtubule asters and its premature interaction with APC/C.	Ribbeck et al. (2006), Song and Rape (2010)
Importin beta-1	BUB3/BuGZ	NLS	Importin beta-1 prevents the premature interaction of BUB3/BuGZ with Ubr5.	Jiang et al. (2015)
Importin beta-1	RepoMan	NLS	RepoMan/importin beta-1 complex localizes to the chromosome periphery at mitotic exit to initiate NE reassembly.	Vagnarelli et al. (2011)
Importin beta-1	PP2A/R1A/B55alpha	NLS	Importin beta-1 and PP2A co-purify and their co-depletion synergistically delays mitotic exit.	Schmitz et al. (2010)
Ciliogenesis and ciliary transport				
Importin beta-1	Crumbs3 (CRB3) alternative splicing product (CRB3-CLPI)	Direct interaction, region of interaction not identified	Importin beta-1 is necessary for CRB3-CLPI ciliary targeting. Expression of dominant-negative importin beta-1 phenocopies the effects of CRB3-CLPI knockdown (loss of cilia, centrosomal and spindle abnormalities).	Fan et al. (2007)
TNPO1	RP2 to ciliary basis	NLS and M9	TNPO1 inactivation abolishes RP2 ciliary entry; pathogenetic mutations in RP2 fall in the M9 signal, blocking RP2 interaction with and ciliary delivery by TNPO1.	Hurd et al. (2011)
TNPO1	Fibrocystin, photoreceptor retinol dehydrogenase, rhodopsin, RP2	CLS	TNPO1 interacts with CLSs in ciliary membrane residents; complexes are regulated by GTPase Rab8 for ciliary import; GEF activity of Rab8 disassembles the complexes and releases free cargoes at the ciliary membrane.	Madugula and Lu (2016)
TNPO1	KIF17	CLS	TNPO1 binds the CLS of KIF17 targets it to the cilium; GTP-locked RAN mutant abolishes ciliary entry.	Dishinger et al. (2010)
Importin alpha/beta-1 dimer^b^		classical NLS	Importin alpha/beta-1 binds the NLS of KIF17 required for nuclear import.	Funabashi et al. (2017)
TNPO1	Gli2 transcriptional activator of Hh	PY-type NLS	TNPO1 regulates ciliary localisation of Gli2; TNPO1 is itself a transcriptional target of Hh, generating a feed forward loop for Gli2 activation in cilia.	Han et. al. (2017)
Importin alpha/beta-1 dimer^b^		classical NLS	Importin alpha/beta-1 binding to NLS is required for Gli2 nuclear import, not for ciliary import.	Torrado et al. (2016)
Modulation / reversal of phase separation of aggregation-prone proteins				
TNPO1	FUS	PY-type NLS	TNPO1 binds FUS and disperses aggregates fromed by intrnsically disordered domains. NLS mutations in FUS prevent TNPO1 binding and aggregate dispersal.	Dormann et al. (2010), Yoshizawa et al. (2018)
Importin alpha/beta-1 dimer	TDP-43	NLS	Importin alpha/beta-1 binding to NLS of TDP-43 disrupts its dimerisation interface and prevents the formation of aggregates.	Guo et al. (2018), Doll et al. (2022)
TNPO1	RNA-binding proteins (RBPs) hnRNPA1, hnRNPA2, FUS, TDP-43	PY-NLS-type	TNPO1 engages th PY-NLSs in hnRNPA1 and hnRNPA2, reversing their fibrillisation. TNPO1 dissolves phase-separation of hydrogels formed by FUS and hnRNPA1. In vivo TNPO1 prevents the accumulation of PY-NLS-containing RBPs (TDP-43, FUS, hnRNPA1, hnRNPA2) in stress granules, restores RBP nuclear localisation, and rescues degeneration caused by disease-linked FUS and hnRNPA2.	Guo et al. (2018)
Importin beta-1 and importin alpha/beta-1 dimer	C9ORF72	Arginine-containing dipeptide repeats (R-DPRs) may act as non canonical importin beta-1 binding sites	Importin beta-1 binds R-DPRs, which induce protein aggregation. This binding interferes with cargo loading onto importins, impairing nuclear import (including of TDP-43). R-DPRs promote phase separation and insolubility of TDP-43, which is suppressed by elevating importin concentrations.	Hayes et al. (2020), Hutten et al. (2020), Nanaura et al. (2021)
Importin alpha-7	hnRNP R, hnRNP U	Bipartite NLS in hnRNP R and monopartite NLS in hnRNP U	Epilepsy-associated E344Q mutation in importin alpha-7 reduces its binding to NLSs, limiting importin alpha-7 binding to both hnRNP R and hnRNP U in iPSCs-derived neurons. Mutations in the hnRNP R bipartite NLS are associated with brain and cerebellar abnormalities.	Oostdyk et al. (2020)

^a^ Only SAFs regulated by NIRs in late mitosis are listed. For NIR-regulated SAFs during mitosis proper see Forbes et al., 2015; Cavazza and Vernos, 2016

^b^ NIRs responsible for nuclear import can be genetically separated from those responsible for ciliary transport.

In late mitosis, importin beta-1 binds the scaffolding protein RepoMan in the N-terminal domain, which targets it around chromosomes, where NE reassembly initiates. Concomitantly, RepoMan binds PP1 *via* its C-terminal domain and also carries PP1 to the chromosome periphery to activate histone dephosphorylation (Vagnarelli et al., 2011), thus coupling chromatin remodelling and NE reorganisation. RepoMan inactivation impairs importin beta-1 recruitment at the nuclear rim, compromising the NE reformation. Importin beta-1 was also identified in a screening for mitotic exit regulators for co-purifying with PP2A/R1A/B55alpha complexes. These complexes have phosphatase activity over proteins previously phosphorylated by the mitotic Cdk1 kinase; their dephosphorylation is needed to complete NE reassembly and chromatin decondensation. Co-depletion of importin beta-1 and PP2A synergistically delays mitotic exit, demonstrating that they cooperate in post-mitotic reorganisation (Schmitz et al., 2010).

In summary, NIRs regulate the localisation:function relationship for factors acting in mitotic progression and exit. Overexpressed NIRs bypass modulation by RANGTP, preventing the release of active factors and disrupting the cell division steps in which they act, originating genetically unbalanced daughter cells that may initiate genomic instability, a cancer hallmark. Indeed, NIRs are overexpressed in many cancer types that display genetic instability and aneuploidy (Çağatay and Chook, 2018).

## Nuclear import receptors shield cargoes from modifying factors

NIRs can modulate the accessibility of their interacting cargoes to external factors. As mitosis progresses towards completion, the anaphase-promoting complex (APC), the major mitotic ubiquitin ligase, acts in two waves: at metaphase completion, it ubiquitinates proteins that must be eliminated to enable chromosome segregation; at mitotic exit, it targets factors that would otherwise prevent interphase resetting. This temporal specificity is achieved by sequential binding of the APC/C to coactivators, CDC20 and CDH1, that have distinct windows of activity.

Two SAFS are identified as APC/C substrates (Table 1): Hepatoma Up-Regulated Protein (HURP), a mitotic microtubule stabiliser (Silljé et al., 2006), and Nucleolar and Spindle-Associated Protein 1 (NuSAP1), which promotes aster formation and fiber elongation, respectively regulated by Importin beta-1 and importin 7, also an importin beta family member (Ribbeck et al., 2006). HURP contains close binding sites for importin beta-1 and for APC/C. Importin beta-1 binding allosterically hides the APC/C site, preventing HURP recognition by APC/C. Importin beta-1 also competes with APC/C for NuSAP1 binding, protecting NuSAP1 from ubiquitination (Song and Rape, 2010). Thus, importin beta-1 shields both SAFs from premature ubiquitination. Microtubules also contribute to protect SAFs from ubiquitination. Simultaneously inhibiting importin beta-1 binding, and APC/C activity, impaired spindle function, indicating that importin beta-1 and APC/C cooperate in regulating the turnover of spindle regulators (Song et al., 2014). When importin beta-1 relocalises around the segregating chromosomes at anaphase, the SAFs become accessible to the APC/C and are conveyed towards degradation.

A similar mechanism operates in control of the mitotic checkpoint complex (MCC), which prevents premature chromosome segregation while microtubule attachments are ongoing. The MCC component BUB3, and its chaperone BuGZ, interact with either importin beta-1 or with the Ubr5 ubiquitin ligase. In prometaphase, when the checkpoint is active, importin beta-1 masks the accessibility of BuGZ and BUB3 to Ubr5. When all chromosomes are spindle-attached, highly concentrated RANGTP around chromosomes displaces importin beta-1, facilitating Ubr5 binding to BuGZ and BUB3: both proteins become ubiquitinated, which reduces MCC activity, triggering anaphase (Jiang et al., 2015).

Thus, importin beta-1 temporally regulates the disappearance of specific proteins by shielding them from premature ubiquitination-dependent proteolysis, modulating the timing of entry into the next phase.

## Nuclear import receptors in ciliogenesis

NIRs localise factors to the primary cilium, a microtubule-based organelle required for the establishment of tissue architecture in almost every cell type and capable to integrate developmental and differentiation signals.

Cilia emerge from centrosomes in cells that exit the cell cycle. Centrosomes are composed of a “daughter” (newly duplicated) and a “mother” centriole, endowed with appendages that anchor it to the cell cortex. The mother centriole will form the cilium basal body in post-mitotic cells, originating a microtubule-based axoneme delimited by the ciliary membrane.

Proteomic and *in situ* studies of cilia have identified NIRs, RAN and RAN-binding proteins (Gupta et al., 2015; Arslanhan et al., 2020). Some cilium-associated proteins (Table 1) bear ciliary localisation signals (CLS) with which NIRs interact, sharing similarities with NLSs (Malicki and Avidor-Reiss, 2014). RANGTP accumulates at the basal body during ciliogenesis, where RAN-binding protein 1 (RANBP1), a negative regulator of nucleotide turnover on RAN, co-localises, suggesting a need for RANGTP modulation at cilia (Fan et al., 2011). NUPs also localise to the basal body, and global inhibition of NUP function prevents ciliary import of kinesin KIF17 (Kee et al., 2012). These data suggest that a size exclusion mechanism regulates protein ciliary entry (Kee and Verhey, 2013)*,* and that RANGTP/GDP cycles control the ciliary release of proteins delivered by NIRs. A ciliary protein screening identified several CLS-tagged factors (Table 1), including retinitis pigmentosa 2 (RP2), as cargoes of importin beta-2/transportin-1 (TNPO1) (Madugula and Lu, 2016)*.* RP2 harbours a nuclear transport signal typical of hnRNP proteins, termed M9, with which TNPO1 interacts. Most RP2 retinopathy-causing mutations fall within the M9 motif, preventing TNPO1 interaction and ciliary entry (Hurd et al., 2011).

Cilia concentrate developmental signals, including Hedgehog (Hh). The Gli2 transcriptional activator of Hh accumulates at cilia, dependent on a proline-tyrosine (PY)-NLS signal (Han et al., 2017), an NLS type recognised by TNPO1 (Lee et al., 2006). Gli2 mutations in the PY-NLS, or TNPO1 knockdown, block Gli2 targeting to cilia. Gli2 ciliary entry proved independent on the importin-alpha/beta-1 nuclear localisation pathway, yet still depends on the import machinery, as RANGTP overexpression, which destabilises import complexes reduces Gli2-expressing cilia (Torrado et al., 2016). Thus, TNPO1 regulates Hh signalling by delivering Gli factors to cilia. The TNPO1-encoding gene is a transcriptional target of Hh, defining a feedforward loop for Gli activation at cilia (Han et al., 2017).

Ciliogenesis is intertwined with intraflagellar transport (IFT) pathways that convey proteins into and along cilia and membrane channels to support cilia functions. Two major motor complexes mediate IFT: the heterotrimeric KIF3A/KIF3B/KAP3 complex, and the homodimeric KIF17 motor. NIRs regulate kinesins involved in IFT.

TNPO1 binds kinesin KIF17 *via* a CLS required for ciliary targeting. KIF17 is then released in the cilium*.* That suggests that ciliary entry requires a ciliary-cytoplasmic RAN gradient: TNPO1 cargo binding would take place in the presence of low cytoplasmic RANGTP, yet RANGTP would need be concentrated in cilia in order to disrupt the TNPO1/KIF17 complex and release KIF17. Indeed, expression of GTP-locked RAN in the cytoplasm disfavoured the TNPO1/KIF17 complex assembly and abolished KIF17 ciliary entry (Dishinger et al., 2010).

The other IFT pathway, utilising KIF3A, KIF3B, and KAP3, acts in anterograde transport (cilia base-to-tip). KAP3 has armadillo-repeats with which importin beta-1 interacts and required both for KAP3 nuclear import and ciliary targeting. *Chlamydomonas, a* model system with cilia regenerating capacity, proved informative to disentangle these processes, due to the evolutionary conservation of KAP3: in that system, assays combining importazole (an inhibitor of importin beta1), and cycloheximide (inhibiting ciliogenesis during regeneration), demonstrated the functional separation of ciliary transport and nuclear import of KAP3 (Huang et al., 2021).

In summary, NIRs control both cilia emergence and ciliary transport, with implications in developmental process, as exemplified by *Hh* and *retinite pigmentosa*. Ciliary defects yield complex, multiorgan developmental disorders (ciliopathies). Consequences are particularly severe in the central nervous system, as cilia regulate polarity in neuronal precursors, migration, organisation of layers, axon pathfinding and circuit formation during corticogenesis (Hasenpusch-Theil and Theil, 2021; Wilsch-Bräuninger and Huttner, 2021; Yang et al., 2021)*.* Experimental depletion of NIRs disrupts cilia formation (Hurd et al., 2011; Han et al., 2017). NIR underexpression during early embryogenesis may therefore be unviable.

## Nuclear import receptors prevent aberrant phase separation of aggregation-prone proteins in neurons

NIRs are global regulators of macromolecular transport in neurons, including retrograde transport, which this highly polarized cells use to transport signalling molecules from peripheral sites in axons into the nucleus of neurons; in that system, cytoplasmic pools of NIRs are poised to carry cargoes over a long distance (reviewed by Rishal and Fainzilber, 2014; Ferreira, 2019). NIRs exert additional roles in neurons, *via* their protein-dispersing ability and capacity to modulate phase separation of protein aggregates. We focus on NIR cargoes found to be mutated in amyotrophic lateral sclerosis (ALS) and frontotemporal dementia (FTD) and summarise how NIRs mitigate the pathogenetic effects of these mutations. NIR functions on other cargoes also relevant to other neurodegenerative forms are listed in (Table 1).

Phase separation is a biophysical transition in which crucial activities concentrate at membraneless subcellular structures or organelles. It can be beneficial when concentrating activities for a specific biological scope, yet can be detrimental when deregulated.

The RNA-binding proteins (RBPs) FUS and TDP-43 harbour intrinsically disordered prion-like domain (PrLD) that can aggregate and undergo aberrant phase transition, associated with progressive neurodegeneration, as in ALS and FTD. These RBPs also contain NIR-interacting signal sequences: FUS bears a PY-NLS recognised by importin beta-2/TNPO1, and this binding not only enables FUS import in nuclei, but also disperses FUS cytoplasmic aggregates (Guo et al., 2018; Yoshizawa et al., 2018). Most ALS-causing FUS mutations fall in the PY-NLS, reducing PY-NLS affinity for TNPO1, with a consequent failure of nuclear import and the concomitant formation of toxic cytoplasmic aggregates (Zhang and Chook, 2012).

TDP-43 is also prone to form aggregates that can be solubilised by importin alpha/importin beta-1 binding to its bipartite NLS (Doll et al., 2022). TDP-43 mutations, also found in a proportion of ALS and FTD cases, yield cytoplasmic aggregates that mislocalise nucleo-cytoplasmic regulators, hence amplifying nuclear transport defects (Chou et al., 2018; Gasset-Rosa et al., 2019).

The C9ORF72 protein, when mutated, actually represents the most frequent cause of ALS and FTD, *via* many sophisticated mechanisms. The common theme in its pathogenetic mechanisms is the expansion of nucleotide repeats in an intron of the primary transcript, which accumulates in part in RNA foci, and in part is aberrantly translated (*via* non-AUG translation) into arginine-rich dipeptide repeat (R-DPRs) proteins. The R-DPRs present in mutant C9ORF72 generate aggregates that interfere with NIR binding to cargoes, and can also “plug” the NPC channel, hence blocking nuclear transport altogether, including wild-type TDP-43 which is found in the cytoplasm. Additionally, the repeats are transcribed in RNA that can bind to and sequester RAN regulators, which also disrupt nuclear import (Hayes et al., 2020). All C9ORF72 pathogenetic mechanisms are mitigated by overexpressing NIRs (Hutten et al., 2020), or NUPs (Freibaum et al., 2015), or by administering nuclear export inhibitors (Zhang et al., 2015).

In summary, NIRs binding to the NLS of proteins carrying aggregation-protein domains prevent aggregate formation, reversing phase separation properties and toxic effects. This capacity of NIRs is linked to both the recognition of NLSs, providing “entry points” for NIRs, and to their ability to engage regions of cargos *via* their large surfaces, with a mechanism distinct but conceptually reminiscent of that used during passage through the NPC and solubilisation of the hydrogels formed by FG-rich NUPs. These effects are independent on nuclear import. Actually, NLS-bearing RBPs can interact with multiple NIR members, suggesting they can utilise a network of NIRs as either chaperones in the cytoplasm or nuclear import vectors (Baade et al., 2021). The level of NIR expression is therefore crucial to prevent neurodegeneration (Moore et al., 2020; Springhower et al., 2020). Indeed, neurodegenerative disorders often display defective expression of NIRs (Pasha et al., 2021). These observations, together with the finding that increasing NIR levels dissolves protein aggregates and reverses their toxicity are raising interest for their potential to prevent or mitigate certain forms of neurodegeneration (Guo et al., 2019; Yoshizawa and Guo, 2021; Odeh et al., 2022).

## Conclusion

Several processes of cellular reorganisation re-adapt NIR-dependent mechanisms operating in nuclear import. In mitosis, NIRs regulate spindle function *via* spatial control of their cargoes. They also regulate the accessibility of cargoes to modifying factors, shielding them from premature phosphorylation and ubiquitination. Roles are also emerging in ciliogenesis. Finally, NIRs can modulate phase separation of aberrant protein aggregates.

**FIGURE 1:**
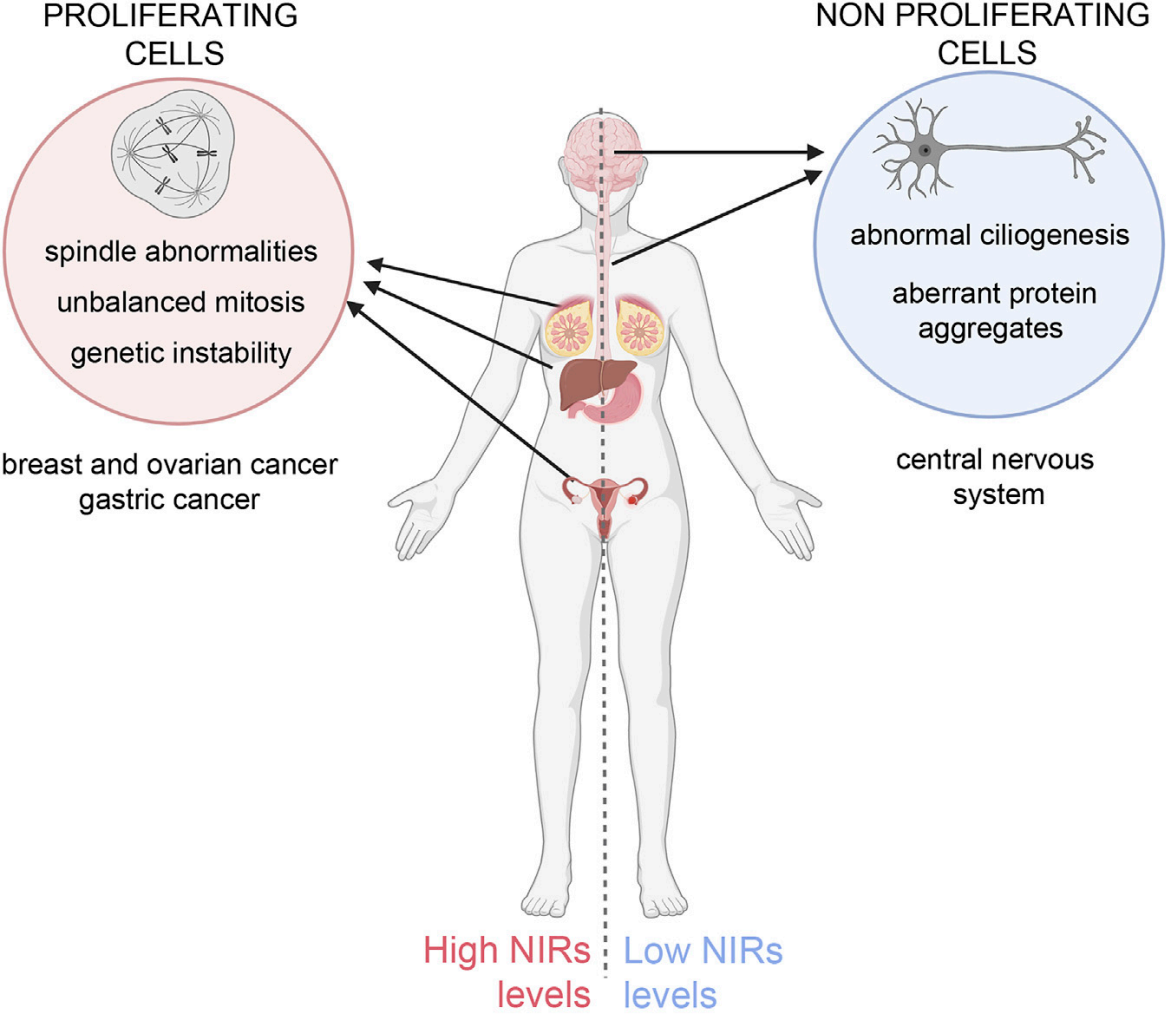
Unbalanced NIRs levels differentially affect cycling and non-proliferating cells. Left panel: overexpression of NIRs in proliferating cells causes mitotic abnormalities that can originate genetic instability, predisposing the cells to neoplastic growth. Indeed NIRs are found to be overexpressed in many tumour types, notably in breast, ovary, hepatocellular and gastric cancer (Çağatay and Chook, 2018; also see the TCGA database (https://www.cancer.gov/tcga, https://portal.gdc.cancer.gov/genes/ENSG00000108424). Right panel: in neurons, in which specific mutations in aggregation-prone proteins lead to the formation toxic aggregates that can undergo phase separation, NIRs can prevent or reverse the formation of such aggregates (see text for details) and mitigate their toxic effects, but fail to do so if expressed at insufficient ratios to the mutant proteins. Low levels of NIR can also impact on ciliary formation and function. The figure was created using BioRender.com.

A challenging issue emerges: on the one hand, NIR overexpression in cycling cells inhibits spindle regulatory factors, causing faulty mitosis and genetic instability in daughter cells, predisposing them to cancer; on the other hand, NIR underexpression in neurons may impair ciliogenesis, causing ciliopathies, and fail to counteract toxic protein aggregates, causing neurodegeneration (Figure 1). Taking this into account, the therapeutic design of utilising the “beneficial” effects of NIRs should be carefully balanced against the risk represented by their intrinsic pro-oncogenic potential. Future molecular and structural developments will help mastering the challenges posed by these multifaceted regulators.

## Abbreviations

ALS, amyotrophic lateral sclerosis; CLS, ciliary localisation signals; FG, phenylalanine-glycine; FTD, frontotemporal dementia; IFT, intraflagellar transport; NE, nuclear envelope; NIR, nuclear import receptor; NLS, nuclear localisation signal; NPC, nuclear pore complex; NUP, nucleoporin; PrLD, prion-like domain; PY-NLS, proline-tyrosine nuclear localisation signal; RBP, RNA-binding protein; R-DPR, arginine-rich dipeptide repeat; SAF, spindle assembly factor.
